# Classification, diagnosis and clinical strategy of congenital coronary artery disease in children

**DOI:** 10.3389/fped.2023.1132522

**Published:** 2023-03-10

**Authors:** Juan Feng, Jingshu Zhao, Jun Li, Zhenyun Sun, Qiao Li

**Affiliations:** ^1^ Department of Ultrasonography, Shandong Provincial Hospital Affiliated to Shandong First Medical University, Jinan, China; ^2^ Shandong First Medical University & Shandong Academy of Medical Sciences, Jinan, China

**Keywords:** congenital, coronary artery disease, children, anatomy, echocardiography

## Abstract

Some of the congenital coronary artery diseases in children have potential life-threatening complications. In addition to anatomical classification, the peadiatricians should pay more attention to the risk of adverse cardiac events classification; and then, they can eventrually make the personalized guidance suggestions and treatment decisions according to different diseases.

## Introduction

Coronary artery is the arterial blood vessel of coronary circulation, which is used to transport oxygenated blood to myocardium. Coronary artery disease (CAD) includes diseases caused by abnormal anatomy and function of coronary artery in a broad sense. It is well known that CAD in children can be divided into two categories: congenital and acquired. The spectrum of acquired coronary artery disease in children is relatively succinct and it is mainly including Kawasaki disease and early onset coronary artery disease caused by and familial hypercholesterolemia in children. In addition, acquired oronary artery lesions can also be a manifestation of several rheumatologic and infectious diseases (1–3). Congenital coronary artery anomaly (CCAA) in children's coronary artery disease spectrum accounts for a higher proportion than adults, and its incidence rate is not high, about 0.5%–3% reported in the literature, but 20% of the lesions have potential life-threatening complications such as severe myocardial ischemia, infarction, arrhythmia and even sudden death (4). Therefore, it is important to understand and grasp the anatomical and pathophysiological characteristics of CCAA. When pediatricians facing this kind of disease it can help make correct understanding, diagnosis and treatment suggestions, thus reducing the probability of malignant cardiovascular events of adolescents causing by CCAA (5).

### CCAA: anatomical classification and risk stratification classification

Under normal circumstances, left coronary artery (LCA) and right coronary artery (RCA) arise respectively from the left coronary sinus(LCS) and right coronal sinus (RCS) of aorta, then it runs on the surface of the heart and finally ends in the capillary bed of myocardium. As long as the congenital development of any of the above links is abnormal, it is called CCAA.

At present, there is no unified classification standard or guidelines of CCAA in the world can be referred to, scholars from home and abroad have put forward many different classification methods (6). Because some classifications are too obscure and cumbersome, in order to facilitate understanding and mastering, this paper is based on the abnormal opening of coronary artery (coronary ostium anomoulies, COA), abnormal running (coronary course anomoulies, CCA) and termination exception (coronary termination anomoulies, CTA); and then CCAA classified according to the above anomalies. If two or more anomalies exist together, such as opening and running anomalies at the same time, the opening anomaly of the above level is taken as the main classification standard. However, no matter how detailed the classification method is, CCAA can only be classified and analyzed macroscopically, it cannot cover all individual variants with low incidence.

The anatomical classification of CCAA is a reliable reference for its precise treatment. Furthermore, according to the risk of adverse cardiac events CCAA can be separated into three kinds: benign coronary anomalies (BCA), potentially serious coronary anomalies(PSCA), serious coronary anomalies(SCA); which refers to CCAA-BCA, CCAA-PSCA and CCAA-SCA, in order to help clinicians better understand and identify the related diseases. See Table 1 for abbreviations and Table 2 for details (7–18).

**Table 1:** Nonstandard abbreviations and acronyms.

Abbreviation	Key Words in Sentence
CAD	Coronary Artery Disease
CCAA	Congenital Coronary Artery Anomaly
LCA	Left Coronary Artery
RCA	Right Coronary Artery
LCS	Left Coronary Sinus
RCS	Right Coronal Sinus
COA	Coronary Ostium Anomoulies
CCA	Coronary Course Anomoulies
CTA	Coronary Termination Anomoulies
BCA	Benign Coronary Anomalies
PSCA	Potentially Serious Coronary Anomalies
SCA	Serious Coronary Anomalies
ALCAPA	Anomalous Left Coronary Artery from The Pulmonary Artery
ACAOS	Anomalous Origin of The Coronary Artery from The Opposite Sinus of Valsalva
ARCAPA	Anomalous Right Coronary Artery from The Pulmonary Artery
CCTA	Coronary Computed Tomography Angiography
CMRA	Cardiac Magnetic Resonance Angiography
TTE	Transthoracic Echocardiography
IVUS	Intravascular Ultrasound

**Table 2:** Classification of CCAA anatomy and risk degree.

	Anomaly of coronary artery opening	Anomalous course of coronary artery after eruption	Anomaly of coronary artery termination	Clinical hazard
CCAA-COA	1. Abnormal number of coronary artery openings in single sinus More than one coronary artery arise from one sinus (7–9) (single opening/multiple openings)		Not involved	1. Without opening stenosis: BCA
				1. Combined with stenosis of opening: PSCA
	2. Coronary artery originates from contralateral aortic sinus (10)	2. The coronary artery often runs in the aortic wall when it is emitted, and there are the following five running modes after it is emitted: A: Inter-arterial course B: Pre-pulmonic course C: Transseptal course D: Retro-aortic course E: Backward course of heart		2.1: PSCA (especially running A)
	2.1 RCA/branch originates from LCS			2.2: PSCA (especially A running)
	2.2 LCA/branch originates from RCS			2.3: PSCA (especially A running)
	2.3 LCA branch originates from RCS/RCA			2 Other subclasses are basically BCA
	3. Single coronary artery It can originate from LCS, RCS, or aorta high opening (11)			3: PSCA
	4. Single coronary orifice atresia (12)			4: SCA
	5. Coronary artery originates from pulmonary artery			
	5.1 RCA originates from pulmonary artery (13)			5.1: PSCA or SCA
	5.2 LCA originates from pulmonary artery (14)			5.2: SCA
	5.3 Both RCA and LCA originated from pulmonary artery (15)			5.3: SCA
	5.4 RCA/LCA branch originates from pulmonary artery			5.4: PSCA
	6. High coronary orifice The coronary artery is located at the junction or sinus of aortic sinus (16) Aortic wall opening			6: BCA or PSCA
	7. Coronary artery originates from extracardiac blood vessels, such as innominate artery and subclavian artery (17)			7: BCA
	8. Coronary artery stenosis-acute angle emanating from aortic sinus			8: PSCA
CCAA-CCA	Not involved	Repeated coronary artery	Not involved	Except for some mural coronary arteries, which are PSCA, the rest are BCA
		Mural coronary artery (myocardial bridge)		
		Subendocardial course of coronary artery		
		Shepherd's crook course of right coronary artery		
		Accessory coronary artery (18)		
CCAA-CTA	Not involved	Not involved	Coronary artery fistula	The major coronary artery fistula is PSCA or SCA, while the minor coronary artery fistula and other communications are BCA
			Communication with other systemic circulation and pulmonary circulation vessels	
Others	Coronary Arch, Congenital Coronary Artery Aneurysm (Ehlers-Danlos Syndrome, Marfan Syndrome) etc.			Coronary Arch: BCA; Coronary artery aneurysm: SCA or PSCA

Note: LCS, Left coronary sinus; RCS, Right coronal sinus; LCA, Left coronary artery; RCA, Right coronary artery; BCA, Benign Coronary Anomalies, PSCA, Potentially Serious Coronary Anomalies, SCA, Serious Coronary Anomalies.

### CCAA-serious coronary anomalies

Coronary artery abnormalities can lead to obvious myocardial insufficiency or hemodynamic changes, and those that can lead to serious clinical consequences without treatment are seriously harmful CCAA.

#### Single coronary artery atresia

It is a rare disease with an incidence of 0.01%–0.04%. Left main coronary artery atresia is more than right coronary artery atresia. The left coronary artery does not communicate with the aorta, but is absent or echoed by fibrous cords. The anterior descending branch and circumflex branch are filled in reverse through the collateral circulation established with the right coronary artery, and the whole heart is completely supplied by the right coronary artery. Therefore, there is a normal right coronary artery “stealing blood” in the original left coronary artery blood supply area, and there is a hemodynamic change basis of extensive ischemia in the left and right coronary artery blood supply areas of the heart. Patients generally have severe myocardial ischemia, heart failure and even sudden death (19).

#### Left coronary artery or both left and right coronary arteries originate abnormally from pulmonary artery

The anomalous left coronary artery from the pulmonary artery (ALCAPA) accounts for 0.25%–0.5% of congenital heart disease (20). Whether the pulmonary artery pressure is enough to maintain the blood supply from the pulmonary artery to the coronary artery of abnormal origin, or the reverse blood supply from the coronary artery of abnormal origin (coronary steal) after the pulmonary artery pressure drops, there would be the possibility of insufficient coronary blood supply, myocardial infarction, heart failure and even sudden death. The mortality rate of ALCAPA in one year after birth without treatment is as high as 90%. Abnormal origin of double coronary arteries from pulmonary artery is very rare, which has more severe clinical symptoms and worse prognosis than ALCAPA.

#### Some large coronary artery fistulas and huge coronary artery aneurysms

The incidence of coronary artery fistulas is low, about 0.002%, which generally has no obvious symptoms in childhood, and can cause various clinical symptoms with the increase of shunt flow in adulthood (21). Studies have shown that the main pathogenic mechanism of coronary artery fistula with large fistula is blood stealing, and coronary artery blood flow does not directly flow into the fistula position through myocardial capillary bed. In addition, coronary artery aneurysm, thrombosis and coronary artery rupture are also common complications (22, 23). It is noteworthy that although coronary artery fistula mostly occurs as the right coronary artery fistula enters the right ventricle or right atrium, it is more likely that the right coronary artery fistula enters the right or left ventricle will cause a huge coronary artery aneurysm and lead to more serious consequences (24, 25).

### CCAA-potentially serious coronary anomalies

As the name implies, although this group of lesions has the potential risk of myocardial ischemia and even sudden death, it does not necessarily occur or have clinical manifestations (26).

#### Anomalous origin of the coronary artery from the opposite sinus of valsalva (ACAOS) combined with inter-arterial course

**Figure 1:**
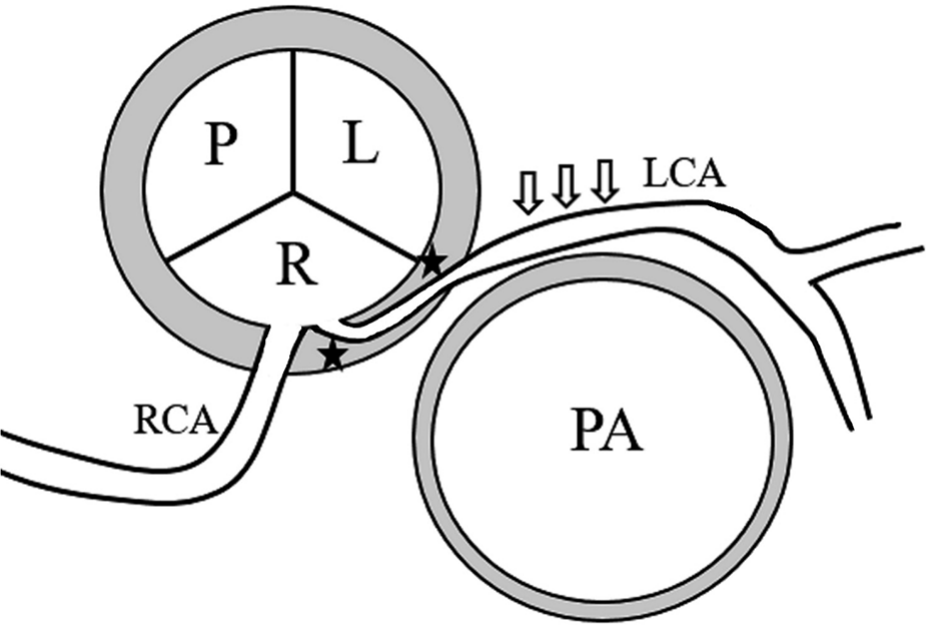
Intermural and inter-arterial course pattern diagram. The anomalous left coronary artery (LCA) from the right coronary sinus with intermural (black star) and interarterial (open arrows) course.

This group of diseases is an important cause of sudden death of teenagers during strenuous activities and military training. The incidence of RCA originating from LCS was 6 times higher than that of LCA originating from RCS (0.15% vs. 0.92%); However, the incidence of LCA originating from RCS in autopsy is much higher than that of RCA originating from LCS, which also suggests that ACAOS in left coronary artery is more likely to be complicated with adverse clinical outcomes (27, 28). Once ACAOS is mentioned, intermural and inter-arterial course are two concepts that must be clarified (Figure 1). When ACAOS occurs, the anomalous originning coronary artery often runs in the aortic wall when it is emitted—this is intermural course. After coronary artery arising from coronary sinus, there are the following five running modes: inter-arterial course, pre-pulmonic course, retro-aortic course, transseptal course and backward couse of heart. Because of the anatomical characteristics of intermural course and inter-arterial course, both of them may be associated with adverse clinical outcomes of ACAOS. Intermural couse means the anomalous coronary artery from the aorta is usually with an acute angle takeoff, which will lead to Slit-like/fish-mouth-shaped orifice. It is postulated that exercise results in the expansion of the aorta, which occludes the acutely angulated slit-like orifice of the coronary artery (29–32). At present, there are many inferences about the mechanism of ACAOS complicated with sudden death during inter-arterial course. The mainstream factors, including proximal coronary artery fissure stenosis, arterial flow compromise, dilated aorta compressing coronary artery during strenuous activity, vasospasm, etc., together lead to the possible manifestations of ACAOS, including not only sudden death, but also dyspnea, palpitation, angina pectoris, dizziness and syncope, and sudden death is usually related to strenuous exercise of young people. Some studies have suggested that the intramural course of coronary artery with abnormal ACAOS is more pathological than the inter-arterial course (33). In addition, some research has shown that transseptal course of an interarterial anomaly can be associated with dysrhythmia. Therefore, so long as there is intramural and/or interarterial course, transseptal course in ACAOS, it may lead to different clinical symptoms and even sudden death in severe cases.

#### Single coronary artery

Only one coronary artery emanates from the aorta and supplies the whole coronary artery tree, and the incidence rate is <0.07% in the population (34). Different from coronary artery occlusion, the structure of a single coronary artery generally has no obvious abnormality, which can originate from LCS or RCS of aorta. Lipton can be divided into three types: L/R I, II and III according to the different origins of the single coronary artery from LCS and RCS and the course of variant vessels (35). The life expectancy of people with single coronary artery is normal, and the incidence probability of coronary atherosclerosis is not higher than that of ordinary normal people. However, a few scholars believe that the subclass of L/R II B variant vessels running between arteries of single coronary artery may lead to myocardial ischemia. Further more, if the single coronary artery arises from pulmonary trunk or part of the single coronary artery has an intraseptal course, the patient should receive operation to reconstruct and decompress the coronary artery system (36).

#### Anomalous right coronary artery from the pulmonary artery (ARCAPA)

ARCAPA is generally milder than ALCAPA in clinical symptoms, and less than 15% of patients have symptoms before the age of 1. The clinical importance of ARCAPA is less than that of ALCAPA, and the actual incidence of ARCAPA may be higher than 0.002% in literature statistics. In order to improve the long-term prognosis of ARCAPA patients, it is recommended that patients with ARCAPA receive surgical correction (37).

This group of CCAA embodies the high heterogeneity of abnormal coronary artery disease spectrum, and even if it is the same disease, its manifestation and pathophysiological mechanism are highly variable. For example, myocardial bridge is widely regarded as a benign lesion, which often has no clinical symptoms. However, in rare cases, individual myocardial bridges, long-term contraction of myocardium and squeezing coronary artery may lead to coronary dissection, spasm and plaque rupture (38–40). Therefore, when facing CCAA of different patients, we should avoid fixed generalization thinking and emphasize individualized analysis for different patients can obtain the most appropriate judgment and diagnosis and treatment suggestions.

### CCAA-benign coronary anomalies

This category covers more than 80% of CCAA. Although coronary artery development is abnormal, it hardly has serious adverse consequences (4). However, as mentioned above, for example, very few mural coronary arteries can also lead to myocardial infarction, which is very rare and requires drug intervention or intervention and surgical treatment (38). However, most CCCA-BCA can be understood as anatomical variation of coronary artery, which will not cause changes in coronary blood supply and will not cause serious clinical consequences. However, some anatomical variations, such as high coronary artery opening and Shepherd's crook course of right coronary artery, suggesting that cardiovascular variations should be reserved for adjustment of operation techniques during coronary intervention diagnosis, treatment and cardiac surgery.

### Clinical manifestations of coronary artery disease in children

As mentioned above, CCAA in children has a wide spectrum of diseases, and its clinical manifestations are also different and varied. Infants and young children may have difficulty feeding, no weight gain, and may be complicated with heart failure and other related manifestations (4). The general symptoms of older children are not specific, such as dyspnea, palpitation, pain in the anterior chest area, dizziness, syncope, etc., and even some teenagers experience sudden death after strenuous activities as the first symptom.

As long as children with signs of heart failure or children with symptoms of precordial discomfort/atypical chest pain after activity, routine electrocardiogram and transthoracic echocardiography (TTE) screening should be performed. The above two inspections are convenient and inexpensive as well as they can provide corresponding information to clinicians. ST-T wave, heart rhythm and rate, abnormal Q wave, etc., are the contents to be observed in electrocardiogram. For children, if the electrocardiogram has changes such as ischemia and abnormal Q wave, differential diagnosis should take CCAA into account in addition to myocarditis, cardiomyopathy, pericarditis and so on. In addition to the evaluation of cardiac structure and function, TTE examination should also focus on the observation of coronary artery. Echocardiographers should try their best to evaluate the opening, inner diameter and proximal course of LCA and RCA carefully. Of course, if TTE has insufficient information, other imaging examinations should be considered. Only by identifying the coronary artery disease can the paediatrician be encouraged to make the appropriate medical guidance to children suspected of CCAA.

### Imaging examinations of coronary artery

As previously mentioned, when the clinical manifestations of children suggest that the physician needs to evaluate the coronary artery anatomy, the first choice for initial imaging diagnosis is still TTE. Although TTE sometimes fails to show the coronary artery clearly, there are still many indirect signs such as obvious mitral regurgitation without positive reasons, enhanced echo of left ventricular endocardium/papillary muscle, dilatation of inner diameter of single coronary artery, extensive collateral branches of left and right coronary arteries were established, etc.; all above suggest that anatomical abnormalities may exist in coronary artery. In this way, children with highly suspected coronary artery disease can be screened for further computed tomography angiography (CCTA) or Cardiac magnetic resonance angiography (CMRA) for precise diagnosis.

Unlike adults who can perform invasive coronary angiography extensively, the suspicious diagnosis of CCAA in children mainly depends on CCTA and CCTA is currently considered the gold standard (41). Except for unavoidable radiation exposure, CCTA has the advantages of good diagnostic speed and accuracy. CMRA has no radiation, but its spatial resolution is slightly lower than CCTA, and its examination time is slightly longer (42). However, it has the advantage of myocardial imaging that CCTA does not have when children need to investigate coronary artery disease for suspicious myocardial lesions. At the same time, we should pay attention to the application of intravascular ultrasound (IVUS) in children, because it is the first choice to evaluate whether there is ischemia possibility when coronary artery anomalies originate from contralateral aortic sinus.

Furthermore, in uncertain cases, electrocardiogram exercise test and stress TTE can assess inducible myocardial ischemia, and electrocardiographic monitoring systems could detect arrhythmic events (41). Children who have been found to have no obvious symptoms of CCAA by imaging examination should undergo further stress cardiac ECT examination to determine whether there is potential blood supply insufficiency (43). It should be pointed out that negative stress imaging examination cannot completely rule out the possibility of potential ischemia or even more serious cardiovascular events. For CCAA children who have developed any symptoms such as dyspnea after exercise and atypical chest pain, further percutaneous coronary angiography and IVUS examination are needed to determine whether to intervene or not (44).

### CCAA prone to easilly missed diagnosis or misdiagnosis

The first choice for coronary artery examination in children is TTE because of its non-radiation, non-invasiveness, low cost and easy implementation. However, due to the influence of ultrasound image quality and the uneven experience of examiners, some diseases such as coronary artery fistula and coronary artery aneurysm are not difficult to find; but some other CCAA, such as ACAOS and ALCAPA, is still quite challenging. ALCAPA is most easily misdiagnosed as dilated cardiomyopathy and endocardial fibroelastosis (45, 46). Because the pathogenesis of ALCAPA is based on insufficient coronary myocardial blood supply, before the normal coronary artery and abnormal coronary artery establish good collateral circulation, it is characterized by cardiac enlargement and cardiac insufficiency. If ultrasound diagnosis is only satisfied with the immediate phenomenon and ignores the evidence of abnormal coronary artery development, it is very easy to misdiagnose. Other CCAA, such as ACAOS, may also be missed by echocardiography. Some ACAOS have acute severe coronary ischemic attack, its clinical manifestations are very similar to fulminant myocarditis. Even if the rescue is temporarily successful, there is the possibility of sudden death in the later stage. Although it is theoretically that ultrasound should clearly show the LCA and RCA originating from aortic LCS and RCS respectively, various illusions exist in actual examination and misjudgments often occur. Even ultrasound doctors with skillful ultrasound techniques and a lot of diagnostic experience can not guarantee that there are no omissions. Therefore, if the doctor suspect the diagnosis of CCAA but ultrasound can not clearly exhibit the opening of coronary artery, it is necessary to ask for further CCTA or CMRA (42). Specious and ambiguous ultrasonic diagnosis is often the first step to cause clinical misdiagnosis and missed diagnosis.

### Clinical management decision of pediatric CCAA

According to the above classification, patients in CCA-SCA group must receive interventional therapy/surgical correction, otherwise they will be at serious risk of cardiac complications and even sudden death (47, 48). The purpose of operation is to restore the normal blood supply of occluded coronary artery and abnormal origin coronary artery and close the abnormal fistula of coronary artery (49, 50). In recent years, minimally invasive interventional therapy has been able to cure the vast majority of coronary artery fistulas and simplify the treatment process.

**Figure 2:**
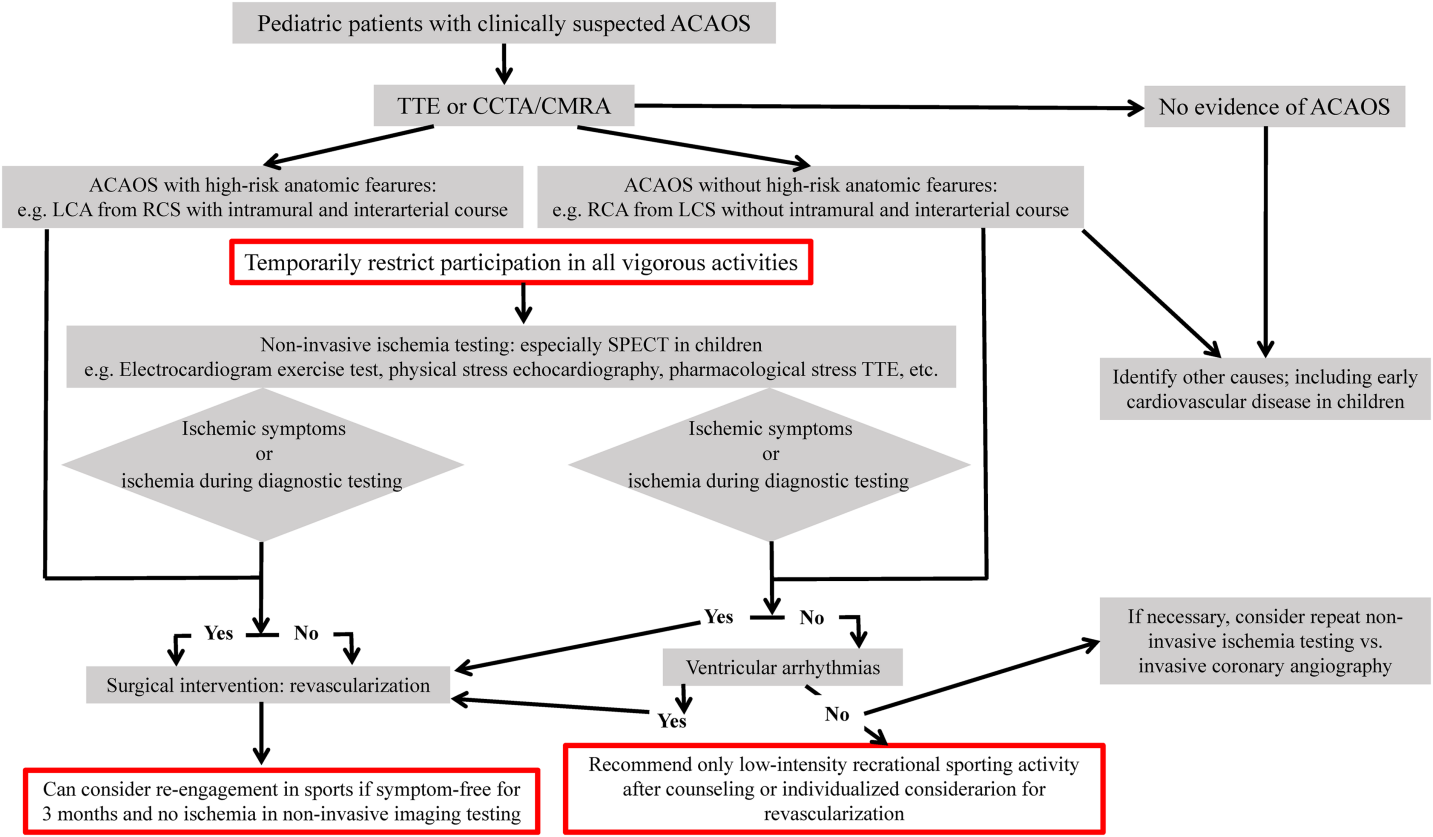
Flow chart for evaluation of clinical strategy of individuals with ACAOS. SPECT, Single photon emission computed tomography; PET, positron emission tomography.

Almost all CCAA-BCA and most CCAA-PSCA do not need special intervention if there is no obvious myocardial ischemia after comprehensive imaging examination. However, if there are potential risks, it is necessary to ask for further multidisciplinary consultation: give medication, put forward suggestions for restricting activities and even necessary intervention and surgical treatment (51, 52). The primary therapy for LCA originating from RCS with interarterial course is surgical reconstruction of coronary blood flow according to the guidelines (4), but the treatment indications are still controversial for asymptomatic children with ACAOS with intramural and inter arterial travel, especially those with RCA from LCS (53, 54). As shown in the flow chart in Figure 2 (55, 56), different diagnosis and treatment suggestions should be made for ACAOS children. The main goal is to prevent sudden death and improve the quality of life. Attention should be paid to the importance of limiting strenuous activities.

## Summary

In general, CCAA should be regarded as an uneven diverse group of congenital disorders whose manifestations and pathophysiological mechanisms are highly variable. The paediatricians should assess the coronary artery lesions accurately, study the clinical manifestations and even potential clinical risks of children, taking into account of the characteristics of children's daily lifestyle; and then, the peadiatricians can eventrually make the personalized guidance suggestions and treatment decisions. During the course of diagnosis and treatment, ECG doctors, cardiac imaging doctors, pediatricians, cardiac surgeons need to work together to develop the most appropriate individualized treatment plan for each child's specific disease.
